# Supplementary material for: Mitotic spindle association of TACC3 requires Aurora‐A‐dependent stabilization of a cryptic α‐helix
Source: EMBO J. 2018 Mar 6;37(8):e97902. doi: 10.15252/embj.201797902 (PMC5897774; doi:10.15252/embj.201797902)
Supplement: Supplementary file 1 — Appendix [file EMBJ-37-e97902-s001.pdf]

APPENDIX

CONTENTS

Figures

Appendix Figure S1. Characterization of genome engineered HeLa cells.

Appendix Figure S2. Purification and structural analysis of ClACCp.

Appendix Figure S3. Electron density maps to support the model for the TACC3 peptide.

Appendix Figure S4. Location of auxilin and pTACC3 in the context of a clathrin cage.

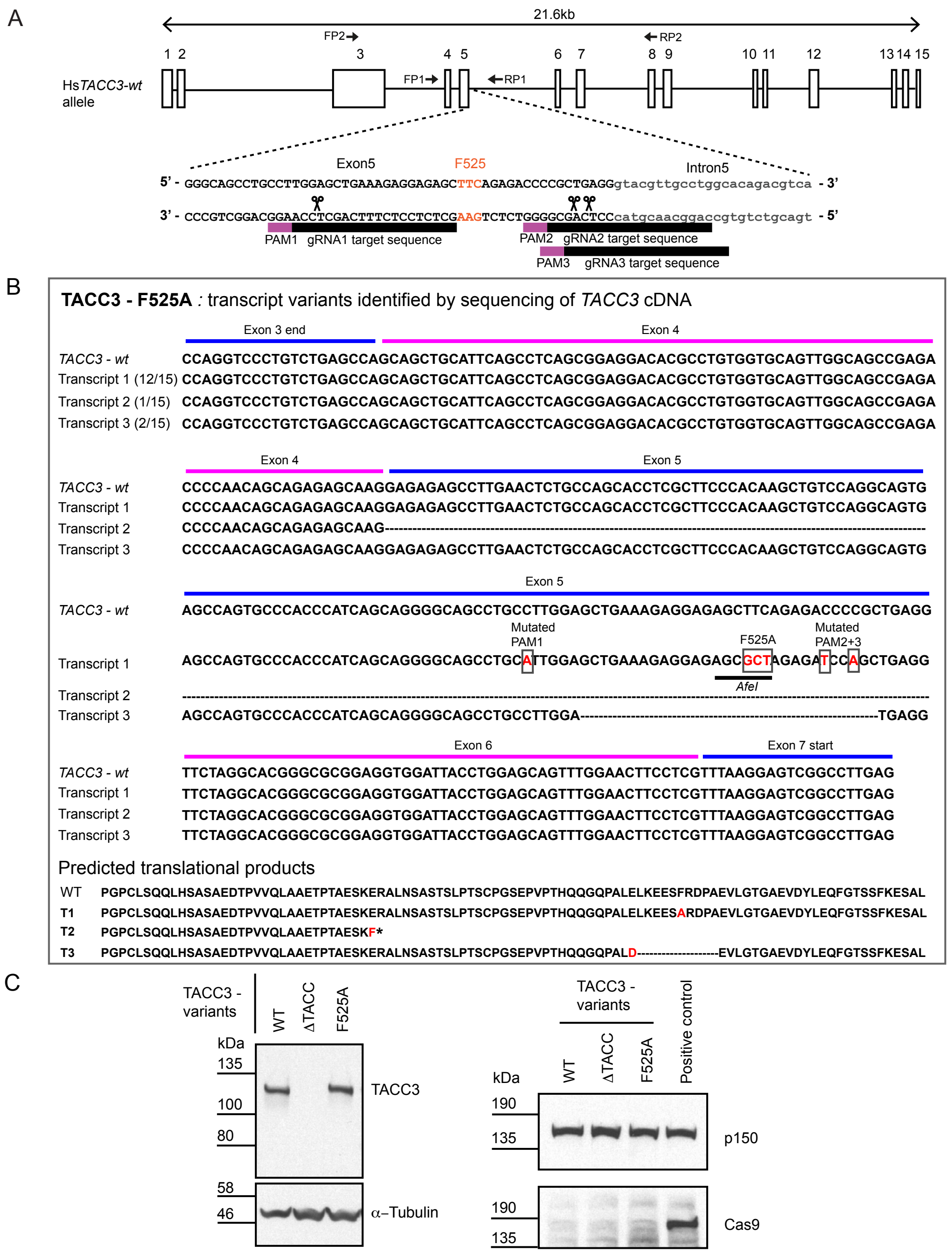
A
21.6kb
FP2
RP2
1 2
3
4 5
6 7
8 9
10 11
12
13 14 15
FP1
RP1
HsTACC3-wt allele
Exon5
F525
Intron5
5' - GGGCAGCCTGCCTTGGAGCTGAAAGAGGAGAGCTTCAGAGACCCCGCTGAGGgtacgttgcctggcacagacgtca - 3'
3' - CCCGTCGGACGGAACCTCGACTTTCTCCTCTCGAAGTCTCTGGGGCGACTCCcatgcaacggaccgtgtctgcagt - 5'
PAM1 gRNA1 target sequence
PAM2 gRNA2 target sequence
PAM3 gRNA3 target sequence
B
TACC3 - F525A : transcript variants identified by sequencing of TACC3 cDNA
Exon 3 end
Exon 4
TACC3 - wt CCAGGTCCCTGTCTGAGCCAGCAGCTGCATTCAGCCTCAGCGGAGGACACGCCTGTGGTGCAGTTGGCAGCCGAGA
Transcript 1 (12/15) CCAGGTCCCTGTCTGAGCCAGCAGCTGCATTCAGCCTCAGCGGAGGACACGCCTGTGGTGCAGTTGGCAGCCGAGA
Transcript 2 (1/15) CCAGGTCCCTGTCTGAGCCAGCAGCTGCATTCAGCCTCAGCGGAGGACACGCCTGTGGTGCAGTTGGCAGCCGAGA
Transcript 3 (2/15) CCAGGTCCCTGTCTGAGCCAGCAGCTGCATTCAGCCTCAGCGGAGGACACGCCTGTGGTGCAGTTGGCAGCCGAGA
Exon 4
Exon 5
TACC3 - wt CCCCAACAGCAGAGAGCAAGGAGAGAGCCTTGAACTCTGCCAGCACCTCGCTTCCCACAAGCTGTCCAGGCAGTG
Transcript 1 CCCCAACAGCAGAGAGCAAGGAGAGAGCCTTGAACTCTGCCAGCACCTCGCTTCCCACAAGCTGTCCAGGCAGTG
Transcript 2 CCCCAACAGCAGAGAGCAAG---
Transcript 3 CCCCAACAGCAGAGAGCAAGGAGAGAGCCTTGAACTCTGCCAGCACCTCGCTTCCCACAAGCTGTCCAGGCAGTG
Exon 5
TACC3 - wt AGCCAGTGCCCACCCATCAGCAGGGGCAGCCTGCCTTGGAGCTGAAAGAGGAGAGCTTCAGAGACCCCGCTGAGG
Mutated PAM1
F525A
Mutated PAM2+3
Transcript 1 AGCCAGTGCCCACCCATCAGCAGGGGCAGCCTGCATTGGAGCTGAAAGAGGAGAGCGCTAGAGATCCAGCTGAGG
AfeI
Transcript 2 ---
Transcript 3 AGCCAGTGCCCACCCATCAGCAGGGGCAGCCTGCCTTGGA-----------------------------------TGAGG
Exon 6
Exon 7 start
TACC3 - wt TTCTAGGCACGGGCGCGGAGGTGGATTACCTGGAGCAGTTTGGAACTTCCTCGTTTAAGGAGTCGGCCTTGAG
Transcript 1 TTCTAGGCACGGGCGCGGAGGTGGATTACCTGGAGCAGTTTGGAACTTCCTCGTTTAAGGAGTCGGCCTTGAG
Transcript 2 TTCTAGGCACGGGCGCGGAGGTGGATTACCTGGAGCAGTTTGGAACTTCCTCGTTTAAGGAGTCGGCCTTGAG
Transcript 3 TTCTAGGCACGGGCGCGGAGGTGGATTACCTGGAGCAGTTTGGAACTTCCTCGTTTAAGGAGTCGGCCTTGAG
Predicted translational products
WT PGPCLSQQLHSASAEDTPVVQLAAETPTAESKERALNSASTSLPTSCPGSEPVPTHQQGQPALELKEESFRDPAEVLGTGAEVDYLEQFGTSSFKESAL
T1 PGPCLSQQLHSASAEDTPVVQLAAETPTAESKERALNSASTSLPTSCPGSEPVPTHQQGQPALELKEESARDPAEVLGTGAEVDYLEQFGTSSFKESAL
T2 PGPCLSQQLHSASAEDTPVVQLAAETPTAESKF*
T3 PGPCLSQQLHSASAEDTPVVQLAAETPTAESKERALNSASTSLPTSCPGSEPVPTHQQGQPALD------------------EVLGTGAEVDYLEQFGTSSFKESAL
C
TACC3 - variants
WT
ΔTACC
F525A
kDa
135
100
80
TACC3
58
46
α–Tubulin
TACC3 - variants
WT
ΔTACC
F525A
Positive control
kDa
190
135
p150
190
135
Cas9

Appendix Figure S1. **Characterization of genome engineered HeLa cells.**

A Strategy for the genome engineering of the *TACC3* locus by CRISPR/Cas9. Location of the guide RNAs and screening primers used for the editing of the *TACC3* locus in HeLa cells.

B Majority of the sequenced bacterial clones (12/15) of cDNA spanning exon3 to exon7 had seamlessly engineered *TACC3* gene with the F525A mutation introduced. However, one clone (1/15) showed the absence of exon 5, resulting in the introduction of a premature stop codon soon after exon4. In addition, two bacterial clones (2/15) had 30 nucleotides missing from the end of exon5 resulting in the deletion of 10 amino acids (which includes F525) with the rest of *TACC3* coding region in-frame. On a western blot, we detected a single band of expected size and no truncated products. Therefore, the majority of TACC3 protein produced is expected to contain the F525A mutations, but a small minority could have a short deletion of 10 amino acids spanning the F525 site. The PAM sites were abrogated by the introduction of silent mutations in the donor oligonucleotide. Deletions are represented by dotted lines. DNA bases or amino acids different from the wild-type sequence are shown in red and the asterisk represents a stop codon.

C Western blot analysis of TACC3 variant HeLa cells. *Left,* TACC3 antibody against aa73-265 fails to recognize a product in ΔTACC, but detects a single band in F525A cells. α-tubulin serves as loading control. *Right,* both ΔTACC and F525A cells do not show stable expression of Cas9. A cell line that stably expresses Cas9 was used as positive control. p150 serves as loading control (left).

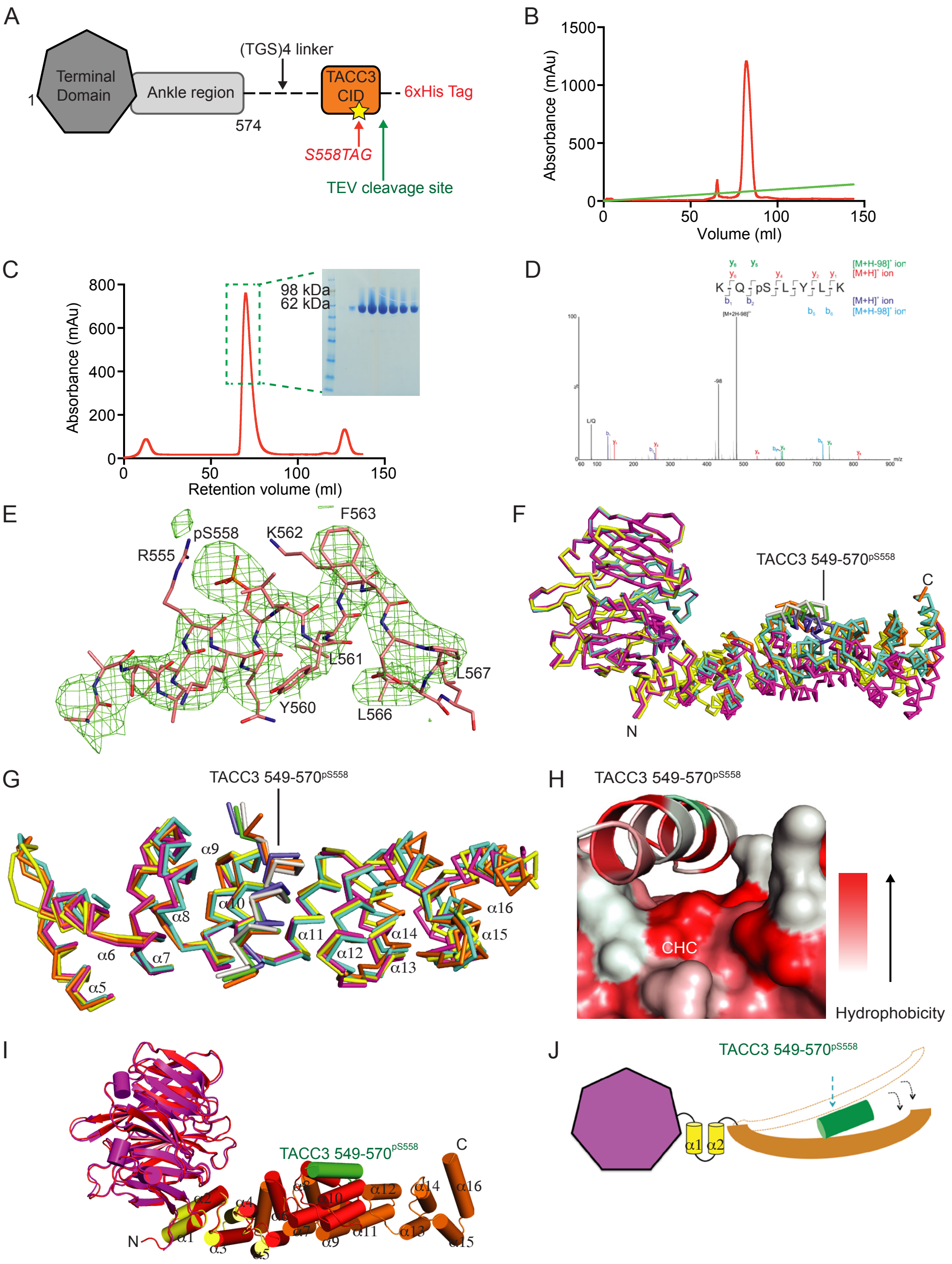
A
Terminal Domain
Ankle region
(TGS)4 linker
TACC3 CID
6xHis Tag
1
574
S558TAG
TEV cleavage site
B
Absorbance (mAu)
Volume (ml)
C
Absorbance (mAu)
Retention volume (ml)
98 kDa
62 kDa
D
E
pS558
K562
F563
R555
L561
L567
Y560
L566
F
TACC3 549-570pS558
C
N
G
TACC3 549-570pS558
H
TACC3 549-570pS558
CHC
Hydrophobicity
I
TACC3 549-570pS558
C
N
J
TACC3 549-570pS558

Appendix Figure S2. Purification and structural analysis of ClACCp.

A Schematic representation of the ClACCp construct used for crystallographic experiments.

B Purification profile of ClACCp on anion exchange chromatography using a HiTrap Q HP 5ml column (GE Healthcare).

C Purification profile of ClACCp by size-exclusion chromatography using a Superdex 200 16/600 column (GE Healthcare). SDS-PAGE analysis of peak fractions on size-exclusion chromatography is shown.

D LC-MS/MS demonstrates the incorporation of phosphoserine at the genetically encoded site (pSer = Ser + 80 Da).

E SA-omit map of $pTACC3^{CID}$ motif contoured at 1 σ. Only few representative residues have been labeled for clarity.

F Superposition of the 4 monomers present in the crystallographic asymmetric unit (ASU) by alignment of CHC β-propeller shows differences in the relative orientations of the helices present in the liker and ankle region in the 4 monomers. These differences arise due to the structural flexibility of the linker and ankle region relative to the TD.

G Superposition of the structures of the ankle region of the four monomers of CHC present in the ASU reveals similar structures of all the four monomers, with a C_α RMSD ranging from 0.701 Å to 1.227 Å. This demonstrates that the mode of interaction of the ankle region with $pTACC3^{CID}$ is equivalent in all the 4 monomers in the ASU.

H The $pTACC3^{CID}$ peptide and its binding surface on CHC 1-574 is color-coded by hydrophobicity. Coloring scheme for the hydrophobicity scale is plotted on the right.

I ClACCp is superposed on apo-CHC 1-494 (PDB 1BPO) by alignment of the CHC β-propeller domains.

J Model showing the mode of interaction of $pTACC3^{CID}$ with CHC 1-574. Based on the structural comparison with apo-CHC 1-494, it is evident that the incoming pTACC3 motif triggers a series of structural rearrangements that is relayed past α1 and α2, with α3- α10 acting as a scaffold that swings and reorganizes to create a binding site for pTACC3.

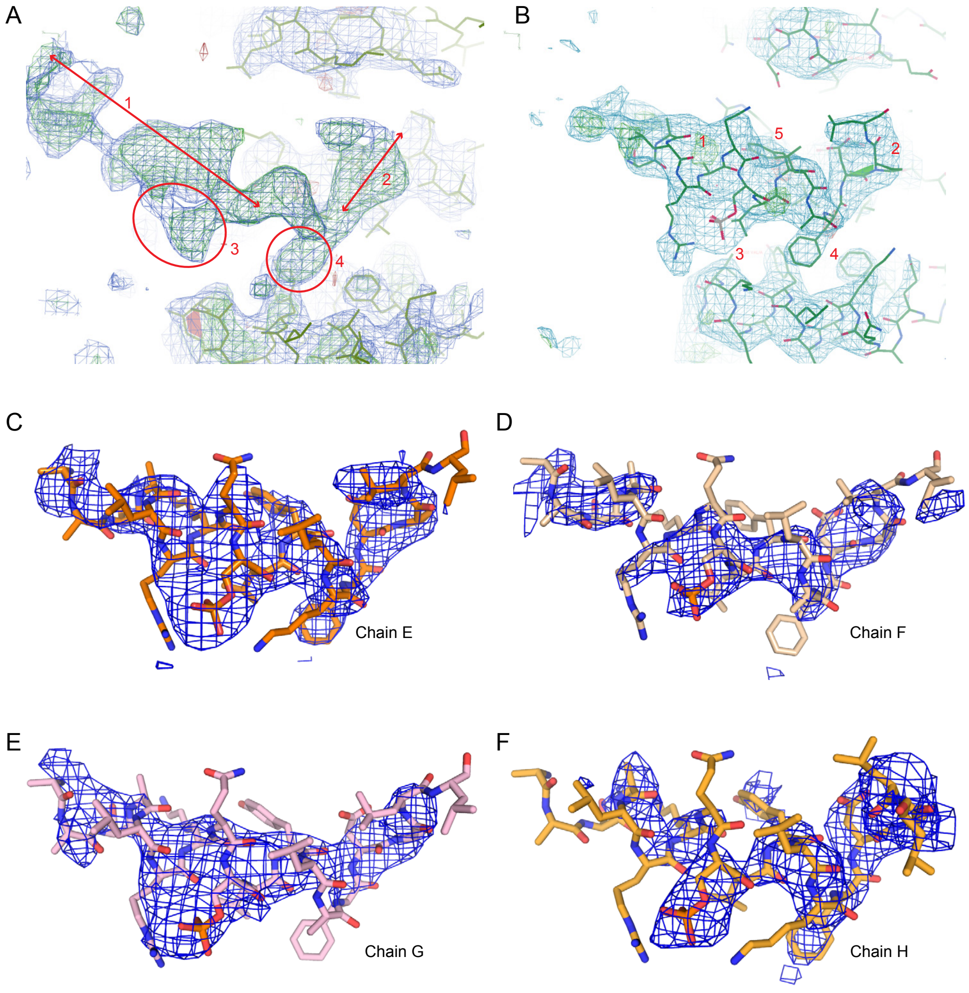
A
1
2
3
4
B
1
5
2
3
4
C
Chain E
D
Chain F
E
Chain G
F
Chain H

Appendix Figure S3 - Electron density maps to support the model for the TACC3 peptide.

A Electron density map at the TACC3 binding site close to chain A of CHC (green lines) after building and initial refinement of the four CHC chains. Maps shown are mFo-DFc (green, red contoured at 2.5 and -2.5 σ, respectively) and 2Fo-DFc (blue, 1.0 σ). Four landmark features of the maps are highlighted in red: 1) a region of α-helix; 2) a region of non-helix, suggesting a very compact turn; 3) a very electron-dense side chain on the α-helix positioned at some distance from the surface of CHC; 4) another electron dense side chain, at the i+5 position relative to the first electron-dense side chain (Y in the figure below).

B TACC3 peptide was built in to the difference density and refined. Additional density corresponding to landmark feature 5 became more prominent after the sidechain of CHC Arg481 was moved to accommodate the sidechain of TACC3 Tyr560.

C-F 2mFo-DFc maps (contoured at 1.0 σ) generated after removal of TACC3 peptide and simulated annealing refinement using PHENIX.

A

B

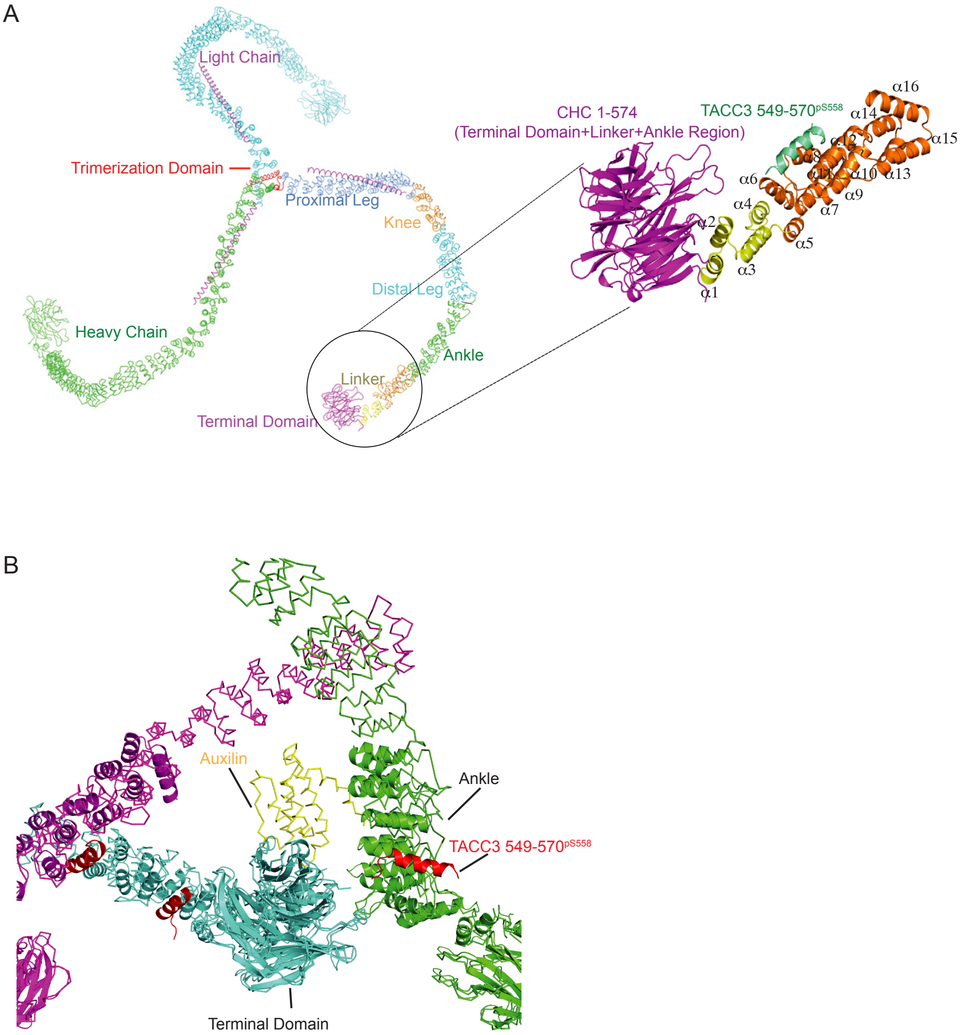

Light Chain
Trimerization Domain
Proximal Leg
Knee
Distal Leg
Heavy Chain
Ankle
Linker
Terminal Domain
CHC 1-574
(Terminal Domain+Linker+Ankle Region)
TACC3 549-570pS558
α1
α2
α3
α4
α5
α6
α7
α9
α10
α13
α14
α15
α16
Auxilin
Ankle
TACC3 549-570pS558
Terminal Domain

Appendix Figure S4 - Location of auxilin and pTACC3 in the context of a clathrin cage.

A Structure of Clathrin triskelion based on PDB 3IYV. The part of the clathrin structure crystallized in this study, CHC 1-574 is encircled.

B Auxilin fits into the intersection of the crossed ankles within the clathrin cage and also contacts the terminal domain emanating from a different hub, based on PDB 1XI5. The ClACCp structure is superimposed by alignment with the corresponding CHC fragments derived from PDB 1XI5.
