# Supplementary material for: Mitotic spindle association of TACC3 requires Aurora‐A‐dependent stabilization of a cryptic α‐helix
Source: EMBO J. 2018 Mar 6;37(8):e97902. doi: 10.15252/embj.201797902 (PMC5897774; doi:10.15252/embj.201797902)
Supplement: Supplementary file 2 — Expanded View Figures PDF [file EMBJ-37-e97902-s002.pdf]

Expanded View Figures

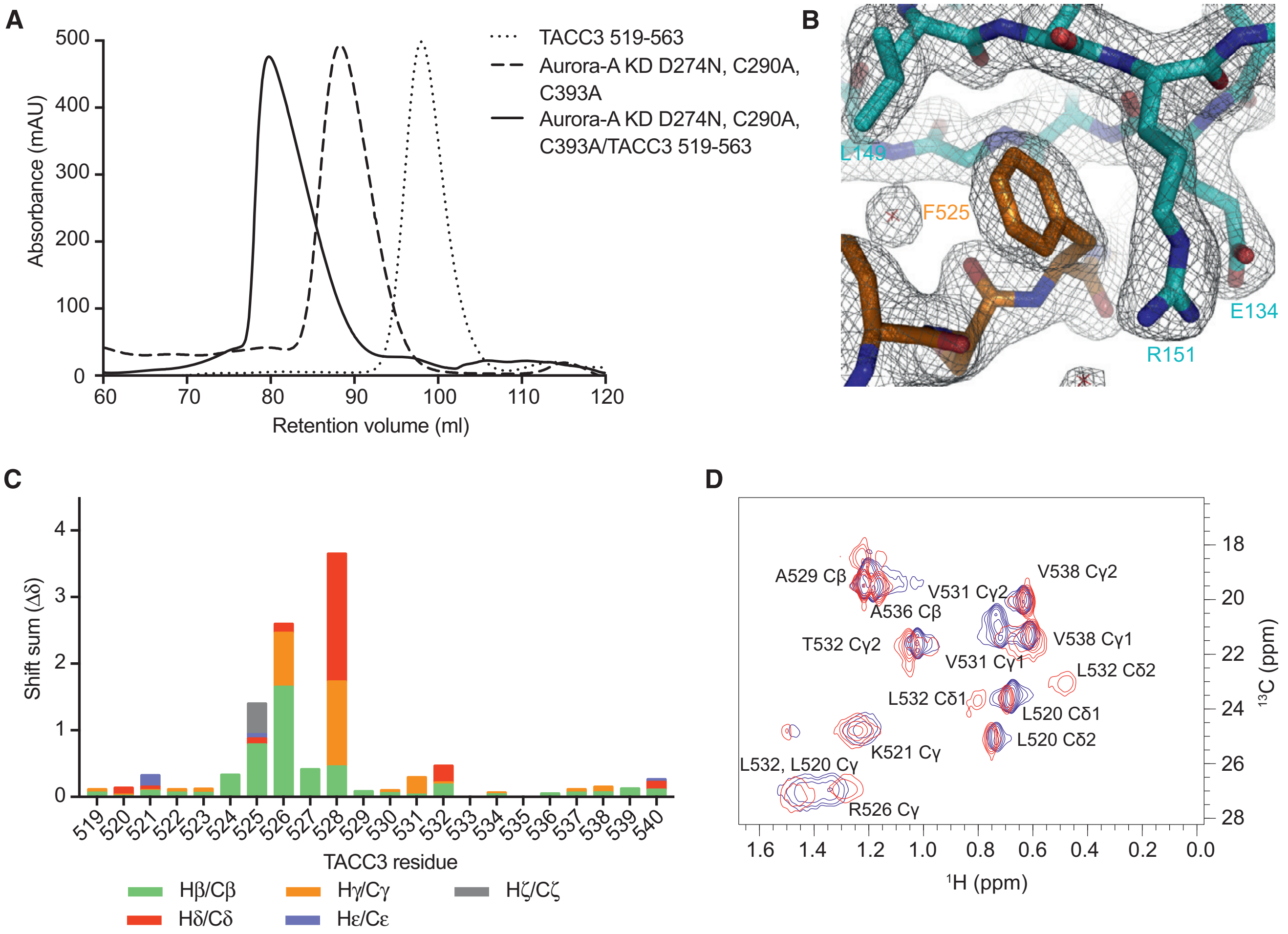

Figure EV1. Structural analysis & size-exclusion chromatography of the Aurora-A/TACC3 complex.

A Size-exclusion chromatographs of Aurora-A and TACC3 alone and in complex on a Superdex 200 16/600 column (GE Healthcare).

B Electron density map of the Aurora-A/TACC3 complex. Aurora-A is coloured cyan. TACC3 is coloured orange. The 2mFo-DFc electron density map is shown as a wire mesh contoured at 1.0 σ.

C Side chain HC chemical shift perturbations observed for $^{13}C^{15}N$ TACC3 519–540 on the addition of Aurora-A^{KD} D274N.

D Small section showing mainly methyl group resonances from a ^{13}C-HSQC spectrum of TACC3 519–540. The spectrum for TACC3 519–540 is shown in blue. The spectrum collected on the addition of Aurora-A^{KD} D274N is coloured red.

© 2018 The Authors

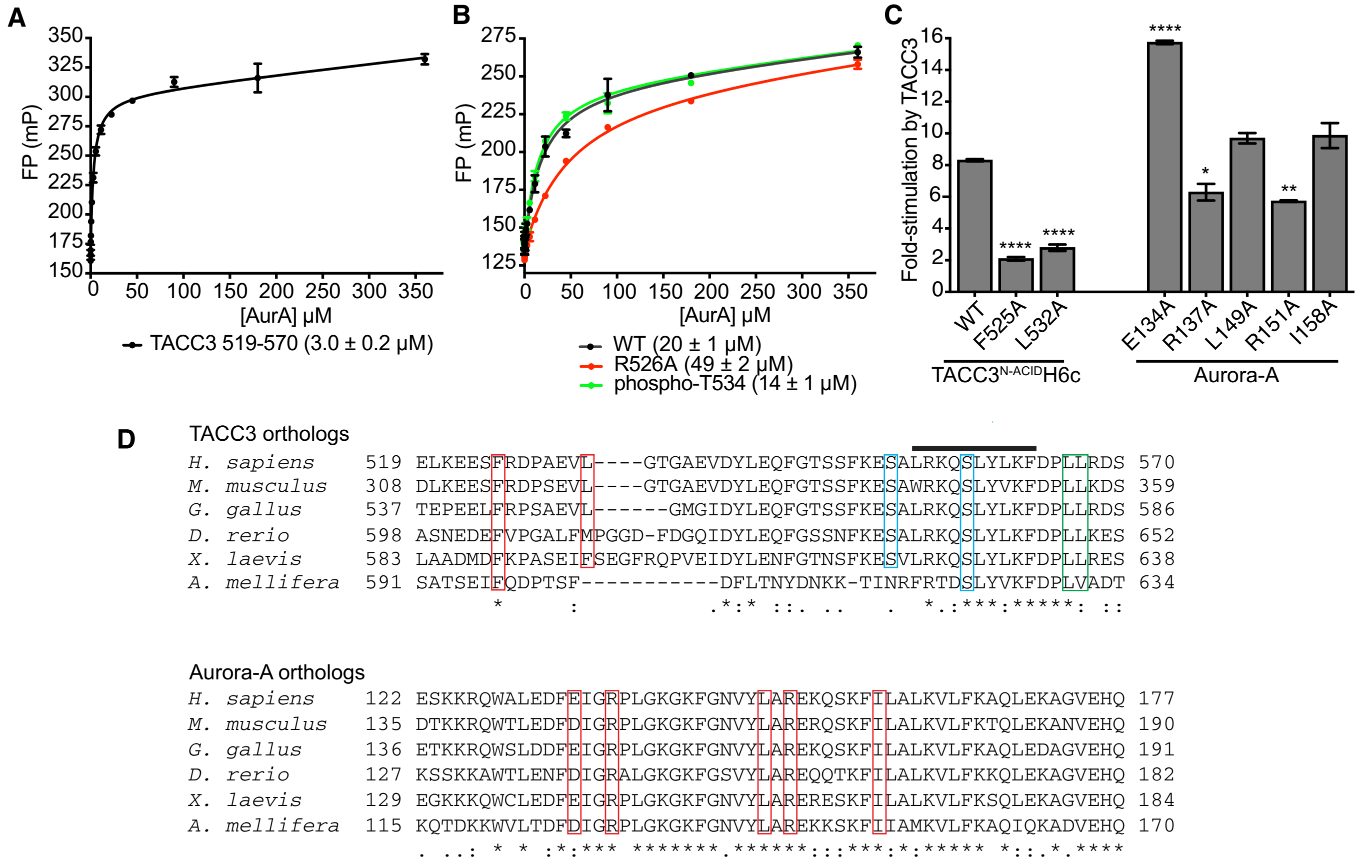

Figure EV2. Biochemical and structural analysis of the Aurora-A/TACC3 complex.

A FP assay between FAM-TACC3 519–570 and Aurora-A. Reactions were performed in triplicate. Error bars represent standard deviation. The binding affinity between WT Aurora-A and FAM-TACC3 519–570 was 3.0 ± 0.2 μM.

B FP assays between wild-type FAM-TACC3 site 1 peptide (residues 522–536) and mutants, and Aurora-A. Reactions were performed in triplicate. Error bars represent standard deviation. The binding affinity between WT Aurora-A and FAM-TACC3 522–536 was 20 ± 1 μM. In contrast, the K_d for the Aurora-A interaction with a FAM-TACC3 522–536 peptide incorporating the R526A mutation was 49 ± 2 μM, a modest increase consistent with the minor contribution of the residue to the interface with Aurora-A. One further peptide, which incorporated phosphorylation of T534, was determined to have a K_d of 14 ± 1 μM. This peptide was based on the location of a sulphate ion, originating from the crystallization solution, at the interface of the Aurora-A/TACC3 interface in the crystal structure, close to TACC3 T534. The modest increase in affinity suggests that this post-translational modification is not significant in regulation of the interaction.

C *In vitro* radioactive kinase assay to determine the effect of TACC3 and phosphorylated Aurora-A mutants on Aurora-A kinase activity. Myelin basic protein (MBP) was used as a generic kinase substrate. Incorporation of radioisotope was measured by scintillation counting. Reactions were performed in duplicate. Error bars represent standard error. Assays were compared to the WT TACC3$^{N\text{-}ACID}$H6c reaction by one-way ANOVA with Dunnett's *post hoc* test. *P < 0.1, **P < 0.01 and ****P < 0.0001.

D Multiple sequence alignment of TACC3 (above) and Aurora-A (below) orthologs spanning the interaction binding interface. Key binding residues are boxed in red. The TACC3 phosphorylation sites, S552 and S558, are boxed in blue. The TACC3 LL motif required for binding to CHC is boxed in green. The TACC3 α-helix produced on binding to CHC is identified by a black line.

 © 2018 The Authors

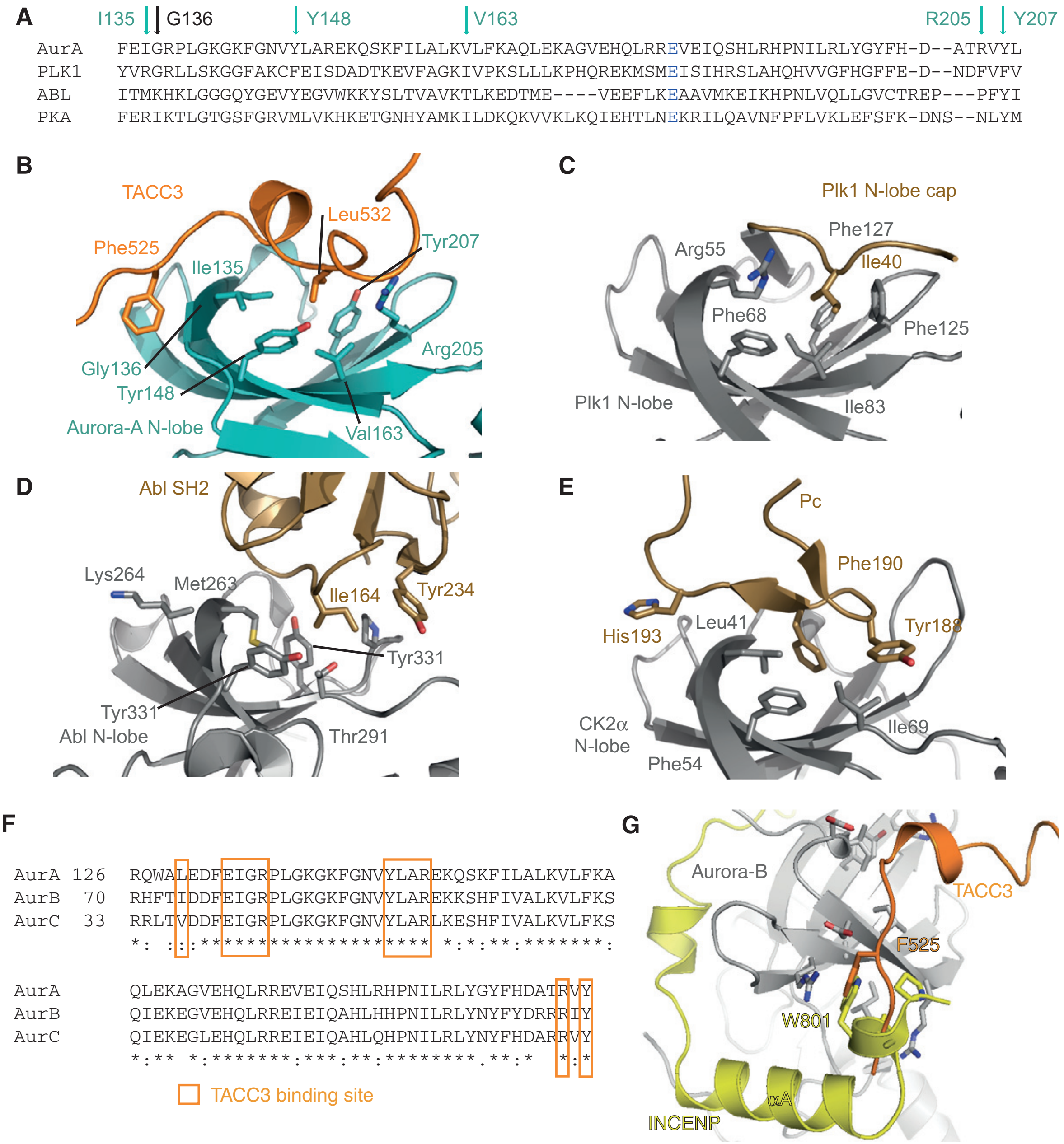

Figure EV3. Structural analysis of the Aurora-A/TACC3 complex.

A Alignment of N-lobe sequences from human kinases Aurora-A, Plk1, Abl and PKA. Arrows mark residues that line the pocket that binds TACC3 L532. Aurora-A E181 is in blue.

B–E Structures of kinase interactions at the N-lobe pocket. (B) Aurora-A (cyan)/TACC3 (orange). (C) Plk1 N-lobe (grey)/cap (beige; PDB 2OU7). (D) Abl N-lobe (grey)/SH2 (beige; PDB 1OPL). (E) CK2α N-lobe (grey)/Pc peptide (beige; PDB 4IB5).

F Alignment of primary sequences from human Aurora-A, Aurora-B and Aurora-C covering the binding site of $TACC3^{N\text{-}ACID}$ on Aurora-A.

G Crystal structure of *Xenopus laevis* Aurora-B (grey)/INCENP (yellow) complex (PDB 2BFX), with TACC3 (orange) superposed.

© 2018 The Authors

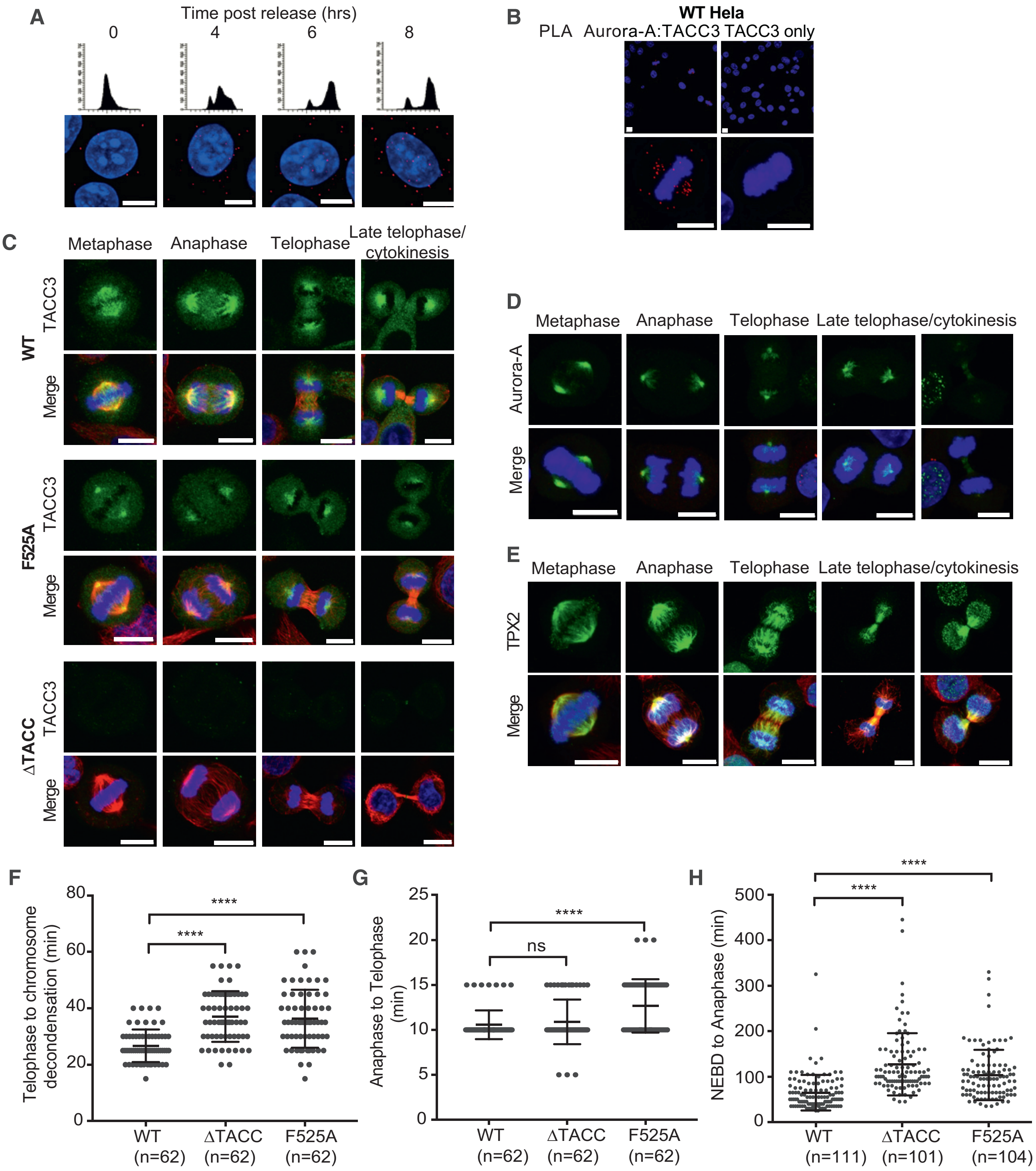

Figure EV4.

© 2018 The Authors

Figure EV4. Analysis of mitotic timings in CRISPR-Cas9 edited cells.

A WT HeLa cells were subjected to a double-thymidine block, released and fixed with ice-cold methanol at time points indicated. Cells were processed for PLA using Aurora-A and TACC3 primary antibodies. FACS profiles show cell cycle distribution, and images below are representative examples of cells from the indicated time point.

B WT HeLa cells were fixed and processed for PLA with Aurora-A and TACC3 antibodies (left) or TACC3 antibody alone (right). Representative images are shown.

C WT, F525A and ΔTACC-HeLa cells were immunostained with TACC3 (Abcam, 1:200, rabbit) antibody followed by incubation with goat anti-rabbit Alexa Fluor 488 (1:200). Note the different pattern of localization of TACC3 in F525A cells compared to WT, both in metaphase and telophase.

D, E WT HeLa cells were immunostained for (D) Aurora-A (Sigma, 1:500, mouse) or (E) TPX2 (Novus biologicals, 1:250, rabbit) (green).

F Duration of telophase to chromosome decondensation, measured in WT, F525A and ΔTACC-HeLa cells by time-lapse microscopy. Number of cells analysed is indicated on graph (*n*).

G Duration of anaphase to telophase measured in WT, F525A and ΔTACC-HeLa cells by time-lapse microscopy. Number of cells analysed is indicated on graph (*n*).

H Duration of NEBD to anaphase, measured in TACC3 variants by time-lapse microscopy. Number of cells analysed is indicated on graph (*n*).

Data information: All scale bars, 10 μm. To assess cell cycle phase, cells in (C–E) were co-stained for either α- or γ-tubulin (Sigma, 1:500, mouse; Sigma, 1:500, rabbit, respectively) (red), and DNA stained with DAPI (blue). Where γ-tubulin co-stain was used instead of α-tubulin, this was due to the α-tubulin rabbit antibody showing weak spindle staining. In the dot plots (F–H), horizontal lines represent the mean and the error bars correspond to standard deviation. *P*-values were obtained from Mann–Whitney: ****$P < 0.0001$; n.s., not significant.

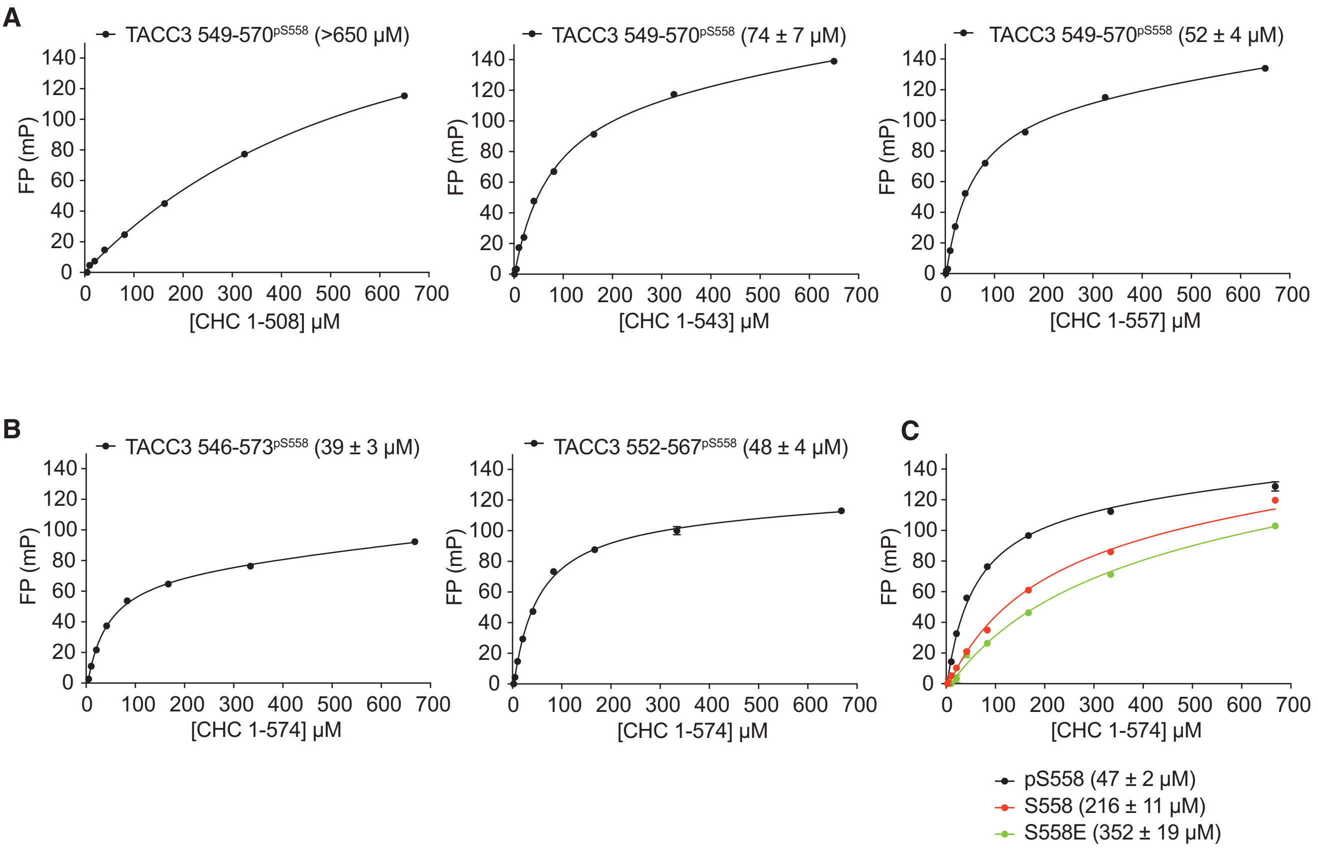

Figure EV5. FP binding data to study the interaction between CHC and TACC3.

A FP assays were performed to measure the binding affinities of four different CHC fragments to FAM-TACC3 549–570 phosphorylated on S558. The longest fragment (aa1–574) had the tightest binding, whereas the shorter fragments showed progressively weaker binding.

B FP assays were carried out to measure the binding affinities of CHC 1–574 with different length FAM-TACC3 peptides phosphorylated on Ser558.

C FP assays to measure the binding affinity of CHC 1–574 to FAM-non-phosphorylated TACC3^{CID} and phosphomimetic S558E TACC3^{CID}.

Data information: Reactions were performed in triplicate with error bars representing standard deviation.
